# Application of Salvage Autologous Blood Transfusion for treating Massive Hemorrhage during Ectopic Pregnancy

**DOI:** 10.3389/fsurg.2022.896526

**Published:** 2022-05-06

**Authors:** Junying Li, Hequn Jin, Zhen Hu

**Affiliations:** Department of Gynecology, Dongyang People’s Hospital, Dongyang, Zhejiang, China

**Keywords:** ectopic pregnancy, massive bleeding, salvage autologous blood transfusion, allogeneic blood transfusion, application value

## Abstract

**Purpose:**

To explore the application value of salvage autologous blood transfusion for massive hemorrhage occurring during ectopic pregnancy.

**Methods:**

A retrospective analysis was performed on the basis of the clinical data of patients in our hospital for the period January 2019 to December 2021. These patients were confirmed to have suffered massive hemorrhage from an ectopic pregnancy during surgery and were treated with blood transfusion. The patients were divided according to their blood transfusion method into three groups: an autologous group (*n* = 46) treated with salvage autologous blood transfusion, a mixed group (*n* = 28) treated with salvage autologous + allogeneic blood transfusion, and an allogeneic group (*n* = 41) treated with allogeneic blood transfusion. The volume of intra-abdominal bleeding, the volume of autologous and allogeneic blood transfusion, postoperative fever and blood transfusion reaction, hemodynamic indices [systolic blood pressure (SBP), diastolic blood pressure (DBP), oxygen saturation (SpO2), and heart rate (HR)] before and after blood transfusion; 24-h postoperative blood routine [hematocrit (HCT), hemoglobin (Hb), platelets (PLT), red blood cells (RBCs)], and electrolyte indices (Na^+^, K^+^, Cl^−^) were all compared among the three groups.

**Results:**

It was found that intra-abdominal bleeding volume in the autologous and mixed groups was higher than that in the allogeneic group (*p *< 0.05), and there was no statistical difference between the autologous and the mixed groups (*p *> 0.05). Autologous blood transfusion volume in the autologous group was higher than that in the mixed group (*p *< 0.05). Allogeneic blood transfusion volume in the allogeneic group was higher than that in the mixed group (*p *< 0.05). After blood transfusion treatment, the postoperative fever rates were 4.35%, 10.71%, and 19.51% in the autologous, mixed, and allogeneic groups, respectively, and the blood transfusion reaction rates were 0.00%, 3.57%, and 9.76%, respectively, which were lower in the autologous group than in the allogeneic group (*p *< 0.05). At 30 min after blood transfusion, SBP, DBP, and SpO_2_ were higher in all three groups than before blood transfusion (*p *< 0.05), and HR was lower than before blood transfusion (*p *< 0.05), but there was no statistically significant difference between the groups at 30 min after blood transfusion (*p *> 0.05). At the 24- h postoperative period, no statistical difference was found when HCT, Hb, PLT, RBC, Na^+^, K^+^, and Cl^−^ were compared among the three groups (*p *> 0.05).

**Conclusion:**

The use of salvage autologous blood transfusion for treating massive hemorrhage occurring during ectopic pregnancy is a safe and feasible method for rescuing patients with such condition because it can rapidly replenish the patient’s blood volume and save blood resources without causing postoperative hemodynamic, blood routine, and electrolyte abnormalities.

## Introduction

Delayed rescue of patients who have suffered massive hemorrhage during ectopic pregnancy can cause shock even death. Timely surgical hemostasis and rapid blood transfusion are the key to successful rescue. However, blood source tension and the spread of AIDS, hepatitis, and other infectious diseases caused by blood transfusion are the two major problems that plague blood transfusion medicine. Internationally, autologous blood transfusion is recommended to address this issue (1, 2). At present, there are three main methods of autologous blood transfusion, namely, prestorage type, dilution type, and recovery type (3). The first two methods are mainly suitable for patients undergoing elective surgery; that is, bloodletting is performed before surgery to prepare for intraoperative or postoperative reinfusion (4, 5). In the third method, the blood that is accumulated in the body cavity or the blood that leaks from the operative area is used as a blood source, which is recovered and processed for transfusion back to the patient (6). It is suitable for patients with acute internal hemorrhage, especially those with massive intra-abdominal hemorrhage who cannot use prestored and diluted autologous blood transfusions due to acute onset and severe condition requiring rapid rescue. It not only saves clinical blood allocation, blood collection, and blood resources and gains resuscitation time for patients, but more importantly, reduces the risk of immune blood transfusion and infectious blood transfusion associated with allogeneic transfusion, which can provide a safer guarantee for patients (7, 8). This study summarizes the experience of the application of intraoperative salvage autologous blood transfusion in patients who suffered massive hemorrhage during ectopic pregnancy in our hospital during the January 2019 to December 2021 period and compares the results with those with the same condition treated with autologous + allogeneic blood transfusion and allogeneic blood transfusion during the same period. The aim of this exercise is to explore the value of salvage autologous blood transfusion in patients who have had massive hemorrhage during ectopic pregnancy. The investigation is reported below.

## Materials and Methods

### Research Object

A retrospective analysis was performed on the basis of the clinical data of patients in our hospital during January 2019 to December 2021. These patients were confirmed to have suffered massive hemorrhage from an ectopic pregnancy during surgery and were treated with blood transfusion. The inclusion criteria were as follows: all patients were diagnosed to have intra-abdominal hemorrhage caused by ectopic pregnancy rupture before operation, all were confirmed to have suffered tubal pregnancy rupture during operation, and postoperative pathological diagnosis confirmed tubal pregnancy; age 18–40 years old; menopause days 35–70 days; urine β-HCG positive; abdominal puncture or vaginal fornix puncture was positive; liver and kidney function and biochemical test results were normal; preoperative blood routine and coagulation function tests were performed; preoperative patients or their relatives signed the “informed consent form for blood transfusion”; case data were complete. The blood transfusion criteria were as follows: the intra-abdominal bleeding time was <24 h, blood was not contaminated, blood was bright red or dark red in color, ectopic pregnancy was <12 weeks, and the fetal membranes were not broken. The exclusion criteria were as follows: hydatidiform mole and choriocarcinoma were excluded, as also the following: previous cardiac, hepatic, renal, hematologic, or immunologic diseases; combined acute and chronic infectious diseases or a recent history of infections; combined severe anemia or coagulation factor deficiency; combined contraindications to blood transfusion; open wound hemorrhage with a duration of more than 4 h; the case data were incomplete. The patients were divided according to their blood transfusion method into three groups: an autologous group (*n* = 46) treated with salvage autologous blood transfusion, a mixed group (*n* = 28) treated with salvage autologous + allogeneic blood transfusion, and an allogeneic group (*n* = 41) treated with allogeneic blood transfusion. There was no statistical difference between the three groups of general data (*p* > 0.05), and they were comparable. See Table 1.

**Table 1:** Comparison of three groups of general data (*n*, $\bar{x}\pm s$).

Group	Autologous group (*n* = 46)	Mixed group (*n* = 28)	Allogeneic group (*n* = 41)	*t*	*p*
Age (years old)	27.59 ± 3.88	26.82 ± 3.77	26.92 ± 3.50	0.509	0.602
BMI (kg/m^2^)	23.08 ± 2.21	23.11 ± 2.19	22.85 ± 1.89	0.177	0.838
Preoperative Hb (g/L)	135.28 ± 6.11	136.25 ± 5.97	136.81 ± 6.12	1.047	0.355
Menopause time (d)	48.39 ± 3.81	49.11 ± 3.86	48.93 ± 4.00	0.510	0.602
Abdominal pain duration (h)	4.74 ± 1.41	4.68 ± 1.44	4.73 ± 1.36	0.012	0.987

### Treatment Method

After a clear diagnosis of intra-abdominal hemorrhage for ectopic pregnancy, treatment such as infusion (crystalloid and colloid) and oxygen inhalation was immediately given, and the necessary preoperative preparations were actively made. After the three parties confirmed the identity of the patient and the surgical site, an emergency surgery was performed under general anesthesia conditions. The patient was placed in a supine position, the surgical field was disinfected with iodophor, a sterile towel was laid on them, which was connected to various parts of the laparoscope, a 10- mm-long skin incision was made on the upper edge of the umbilicus, a pneumoperitoneum needle was punctured, and CO2 gas was charged to make the intra-abdominal pressure reach 13 mmHg, a Trocar with a diameter of 10 mm was placed on them, and a laparoscope was inserted into the abdominal cavity. A 105- mm-diameter Trocar was placed in the left and right lower abdomen under laparoscopic direct vision. An investigation of intra-abdominal hemorrhage and blood clots was done, surgical excision of the lesions was performed, and a suture repair of the bleeding lesions and hemostasis was done.

On this basis, the autologous group adopted salvage autologous blood transfusion: the instrument used was Cell Saver 5+ (National Food and Drug Administration (Jin) Zi 2009 No. 3542073). Anticoagulant (500 mL of saline with 25,000 U of heparin), blood reservoir, suction device, connecting tube, centrifugal pump, and flushing solution were made ready just before the operation. Blood was collected at the beginning of the surgery by using an aspirator to draw blood into the blood reservoir, and blood resulting from traumatic bleeding, intra-abdominal bleeding, and intraoperative irrigation fluid was also collected. The blood was filtered through multiple layers of the blood storage tank. When the blood storage volume reached 500–600 mL, the automatic processing system of centrifugation, cleaning, and purification was activated. After cleaning, the anticoagulant, tissue debris, free hemoglobin, and anticoagulant were dispensed into the waste bag, and the concentrated red blood cells were drained into the blood bag and directly returned to the patient for use. Allogeneic group transfusion of allogeneic stock blood is explained as supplementation with the appropriate concentrated red blood cell suspension and plasma according to the patient’s blood loss and vital signs. The mixed group was treated with combined autologous + allogeneic blood transfusion.

### Observation Indicator

The volume of intra-abdominal bleeding, autologous and allogeneic blood transfusion, postoperative fever, and blood transfusion reaction were counted in the three groups. The hemodynamic indices of systolic blood pressure (SBP), diastolic blood pressure (DBP), blood oxygen saturation (SpO_2_), and heart rate (HR) were recorded before and 30 min after blood transfusion in the three groups. Blood routine consisting of hematocrit (HCT), hemoglobin (Hb), platelets (PLT), and red blood cells (RBC) was recorded for the 24- h postoperative period in the groups. The electrolyte indices of Na^+^, K^+^, and Cl^−^ were also recorded for the same period.

### Statistical Methods

SPSS 25.0 statistical software package was used to analyze the research data. The normally distributed measurement data were expressed as mean ± standard deviation, the intragroup comparison was performed by using the paired *t*-test, and the multigroup comparison was performed by using variance analysis. Enumeration data were expressed as rates, and comparisons were performed by using *χ*^2^ analysis. A score of *p *< 0.05 indicates that the difference is statistically significant.

## Results

### Comparison of the Volume of Intra-Abdominal Bleeding and Autologous and Allogeneic Blood Transfusion in Three Groups

**Figure 1:**
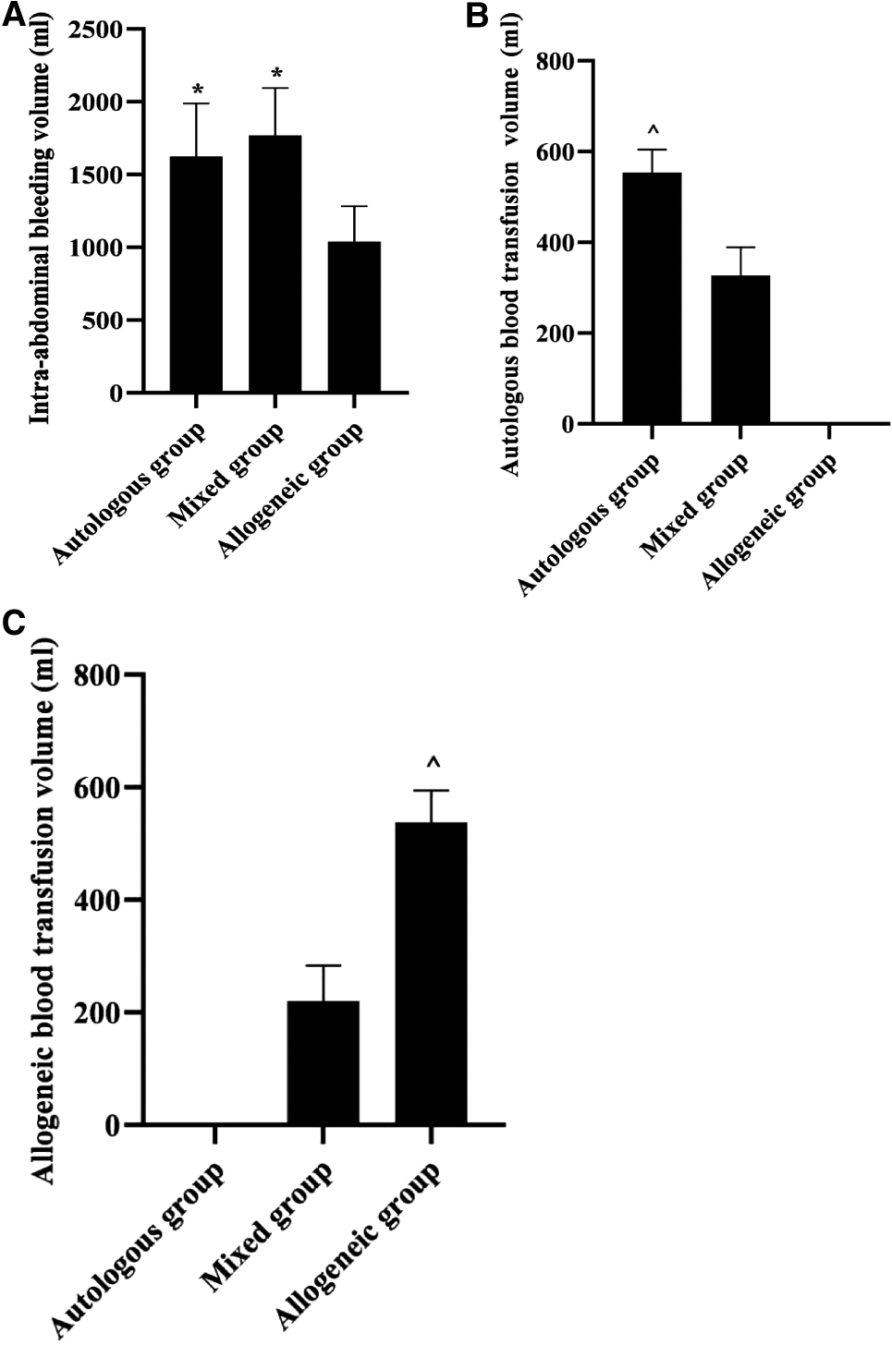
Comparison of the volume of intra-abdominal bleeding and autologous and allogeneic blood transfusion in three groups ($\bar{x}\pm s$, mL). Note: (**A**) Intra-abdominal bleeding volume (mL). (**B**) Autologous blood transfusion volume (mL). (**C**) Allogeneic blood transfusion volume (mL). *p < 0.05 compared with the allogeneic group; ^^^*p *< 0.05 compared with the mixed group.

The results revealed that intra-abdominal bleeding volume in the autologous and mixed groups was higher than that in the allogeneic group (*p *< 0.05), and there was no statistical difference between the autologous and the mixed groups (*p *> 0.05). Autologous blood transfusion volume in the autologous group was higher than that in the mixed group (*p *< 0.05). Allogeneic blood transfusion volume in the allogeneic group was higher than that in the mixed group (*p *< 0.05). See Figure 1.

### Comparison of Postoperative Fever and Blood Transfusion Reaction in Three Groups

**Figure 2:**
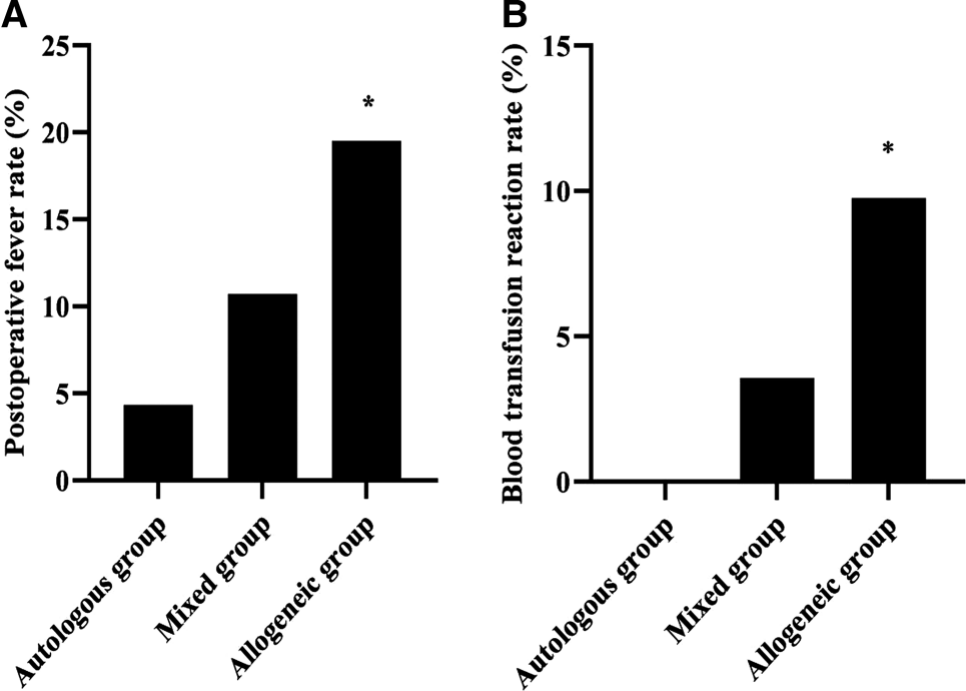
Comparison of postoperative fever and blood transfusion reaction in three groups (*n*, %). Note: (**A**) Postoperative fever rate (%). (**B**) Blood transfusion reaction rate (%). *p < 0.05 compared with the autologous group.

After blood transfusion treatment, the postoperative fever rates were 4.35%, 10.71%, and 19.51% in the autologous, mixed, and allogeneic groups, respectively, and the blood transfusion reaction rates were 0.00%, 3.57%, and 9.76%, respectively, which were lower in the autologous group than in the allogeneic group (*p *< 0.05). See Figure 2.

### Comparison of Hemodynamic Indices Before and After Blood Transfusion in Three Groups

**Figure 3:**
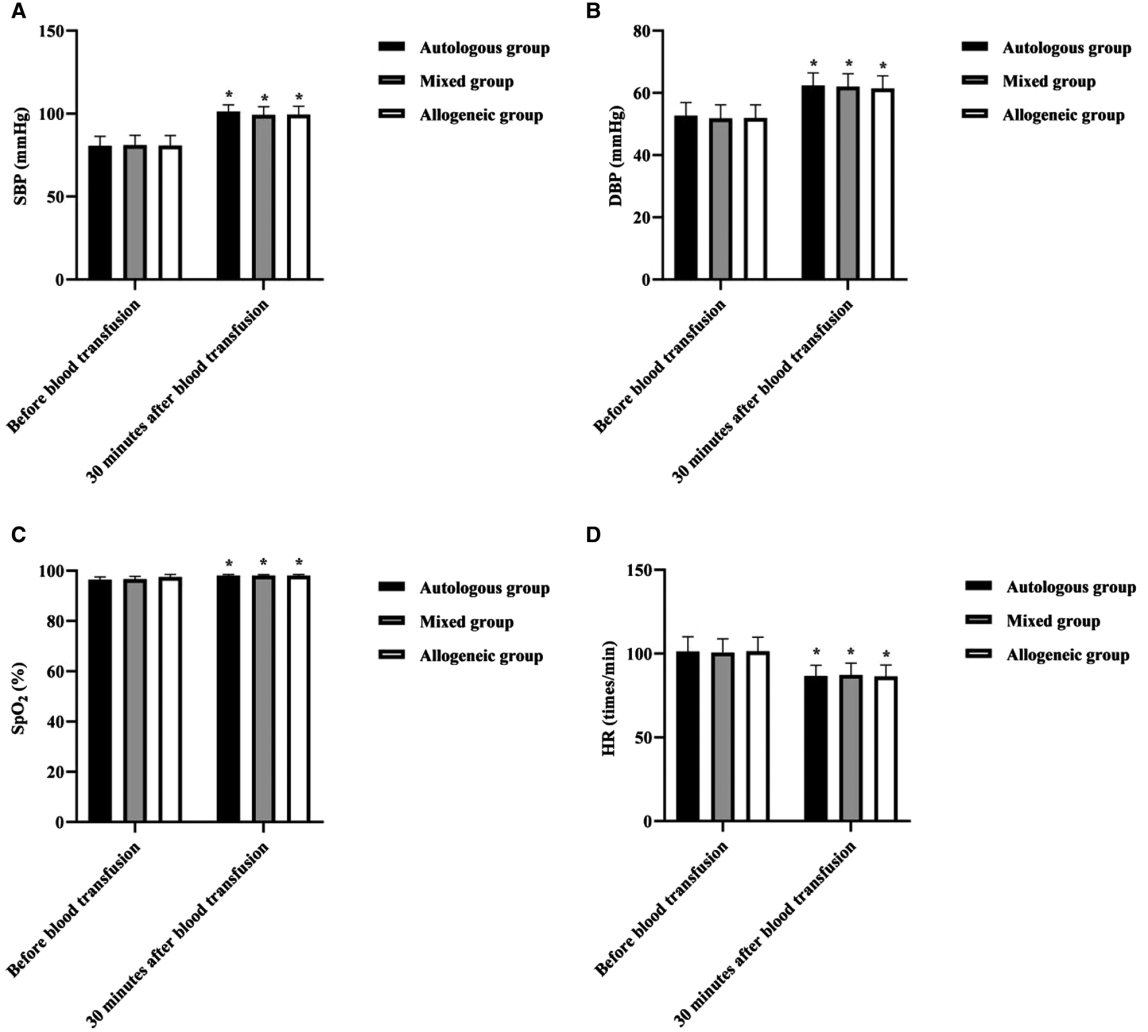
Comparison of hemodynamic indices before and after blood transfusion in three groups (*n*, $\bar{x}\pm s$). Note: (**A**) SBP (mmHg). (**B**) DBP (mmHg). (**C**) SpO_2_ (%). (**D**) HR (beats/min). *p > 0.05 when comparing before and 30 min after blood transfusion in the same group.

At 30 min after blood transfusion, SBP, DBP, and SpO_2_ were higher in all three groups than before blood transfusion (*p *< 0.05), and HR was lower than before blood transfusion (*p *< 0.05), but there was no statistically significant difference between groups at 30 min after blood transfusion (*p *> 0.05). See Figure 3.

### Comparison of 24 h Postoperative Blood Routine in Three Groups

**Figure 4:**
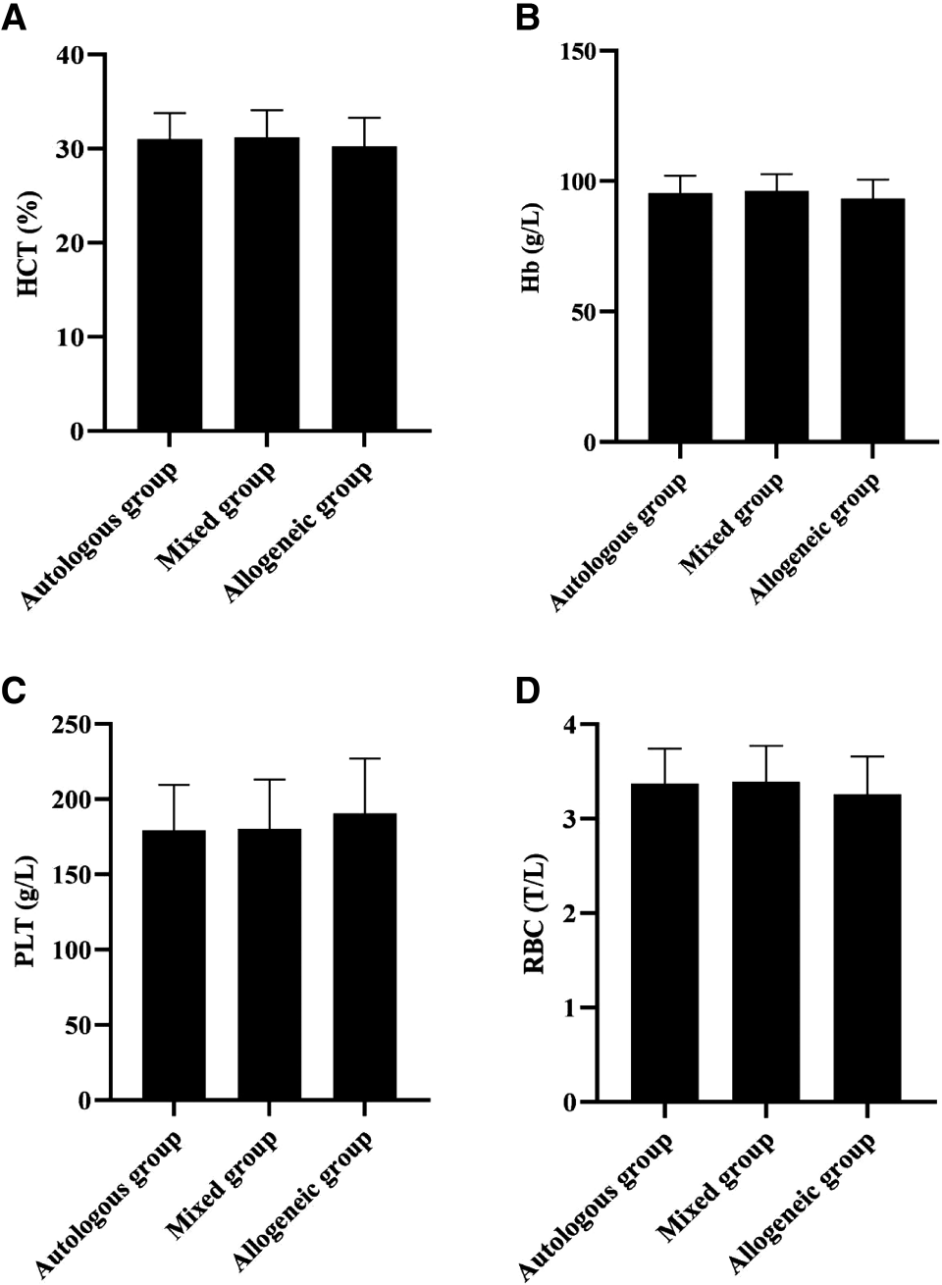
Comparison of 24 h postoperative blood routine in three groups (*n*, $\bar{x}\pm s$). Note: (**A**) HCT (%). (**B**) Hb (g/L). (**C**) PLT (g/L). (**D**) RBC (T/L).

At the 24- h postoperative period, no statistical difference was noted during the comparison of HCT, Hb, PLT, and RBC among the three groups (*p *> 0.05). See Figure 4.

### Comparison of 24 h Postoperative Electrolyte Indices in Three Groups

**Figure 5:**
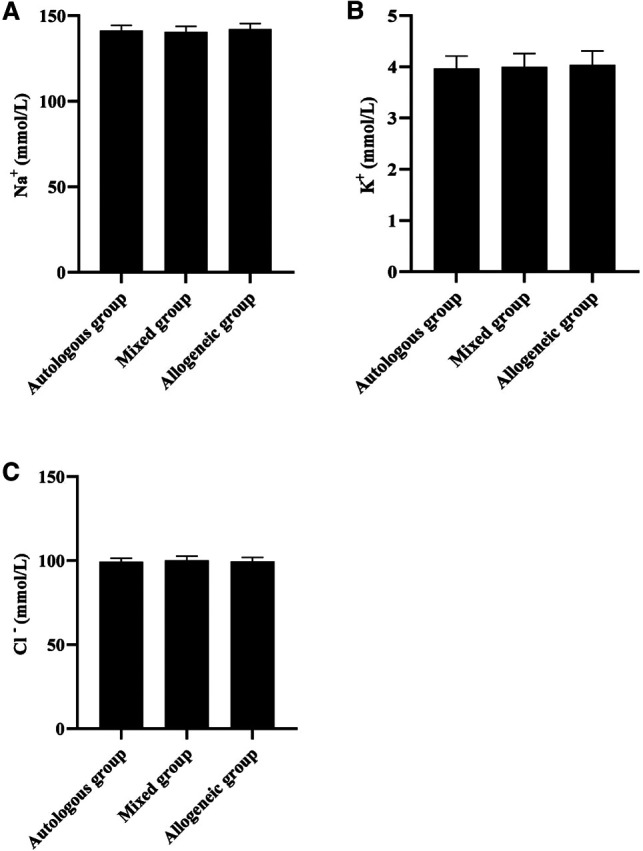
Comparison of 24- h postoperative electrolyte indices in three groups ($\bar{x}\pm s$, mmol/L). Note: (**A**) Na^+^ (mmol/L). (**B**) K^+^ (mmol/L). (**C**) Cl^−^ (mmol/L).

At the 24- h postoperative period, no statistical difference was found during the comparison of Na^+^, K^+^, and Cl^−^ among the three groups (*p *> 0.05). See Figure 5.

## Discussion

Ectopic pregnancy is a common emergency abdominal condition in obstetrics and gynecology but is an emergency with an acute condition and a lot of bleeding, with an incidence of about 2% to 3% (9). It refers to the implantation of a fertilized egg outside the uterine cavity, which, if not treated promptly, can lead to hemorrhagic shock, accounting for about 9% to 13% of pregnancy-related deaths, and requiring surgical hemostasis and blood volume replenishment in the first instance. Blood transfusion is an effective means of treatment for this condition (10). The current blood sources are allogeneic blood and autologous blood. Of these, the transfusion of allogeneic blood requires tedious steps such as matching and transport, which may easily lead to patients missing the golden period of rescue. In addition, the transfusion of allogeneic blood poses risks that are difficult to eliminate, such as allogeneic immunosuppression, transfusion-transmitted diseases, hemolytic reactions to transfusion, and technical errors that may occur during operations such as blood matching, histocompatibility, and disease testing when blood sources are tight and cannot be supplied in sufficient quantities in a timely manner. Autologous blood transfusion has become an important measure in the treatment of ectopic pregnancy bleeding.

Our data showed that intra-abdominal bleeding volume was higher in the autologous and mixed groups than in the allogeneic group (*p *< 0.05), and there was no statistical difference between the autologous and the mixed groups (*p *> 0.05); autologous blood transfusion volume in the autologous group was higher than that in the mixed group (*p *< 0.05); allogeneic blood transfusion volume in the allogeneic group was higher than that in the mixed group (*p *< 0.05). These show that the method of salvage autologous blood transfusion achieves the purpose of reducing blood loss and saving blood resources through the utilization of “waste blood”. According to the briefing, the average blood donation rate per 1,000 people in China has increased from 4.8% in 1998 to 11.1% in 2020, but it is still lower than the average blood donation rate per 1,000 people in high-income countries in the world, which is 45.4%, and meets only the 10% rate recommended by the World Health Organization. Also, the foundation of blood supply, especially non-remunerated blood donation, is still relatively weak in China (11). In contrast, a salvage autologous blood transfusion is a transfusion method that uses a blood recycling machine to return blood accumulated in the body cavity after trauma or blood lost during surgery to the patient themselves after anticoagulation and filtration (12). Concentrated red blood cells with a hematocrit of 50%–65% can be obtained after automatic processing by the blood recovery machine (13). It is efficient and fast, without depending on the blood type and cross-matching results, and requires only a simultaneous recovery of the patient’s intraoperative blood loss at the beginning of the procedure, so that the processed final concentrated red blood cells can be directly transfused back to the intraoperative patient, thus gradually becoming an important blood protection technique for patients with emergency hemorrhage.

Allogeneic blood transfusion has risk factors affecting blood quality and safety in all aspects of blood collection, preparation, testing, storage, transportation, and clinical transfusion. Up to a dozen diseases have been clinically confirmed to be transmitted through blood, and four infectious agents are mainly detected in China: hepatitis B, hepatitis C, syphilis, and AIDS (14). Other unknown blood-borne viruses may also be present in human blood, which can exacerbate the risk of allogeneic transfusions. Transfusion transmission of HIV is currently reported in various countries (15, 16). Due to limited medical technology, the problem of window periods for infectious diseases still plagues the detection of blood-related pathogens. Our data showed that after transfusion therapy, the postoperative fever rates were 4.35%, 10.71%, and 19.51% in the autologous, mixed, and allogeneic groups, respectively, and the transfusion reaction rates were 0.00%, 3.57%, and 9.76%, respectively, all of which were lower in the autologous group than in the allogeneic group (*p *< 0.05). Transfusion reactions mainly manifest as immune transfusion reactions such as chills, itchy facial sensation, and rubella (17, 18). This shows that the salvage autologous blood transfusion effectively avoids the risk of allogeneic blood transfusion to the patient. This may be due to the fact that the blood recovered from autologous blood transfusion is separated by filtration, washing, and centrifugation, which removes villi and embryonic tissue, traumatic tissue debris, clots, contaminants, and destroyed blood cells, plasma viable fractions, free Hb, plasma, fatty acids, and anticoagulants, resulting in concentrated red blood cells (19). Although this method causes some damage to the red blood cells and discards the plasma components, it removes the harmful cell debris and free Hb, and the final red blood cells obtained are of high quality, so there are few adverse reactions after the return transfusion.

The cause of hemorrhagic shock in patients with hemorrhagic ectopic pregnancy is the rapid loss of circulating blood volume resulting in a sharp drop in blood pressure, tissue hypoxia, and increased heart rate (20). Rapid blood volume replacement is the primary measure to correct hemorrhagic shock. Our data showed that 30 min after blood transfusion, SBP, DBP, and SpO2 in all three groups were higher than before blood transfusion (*p *< 0.05), and HR was lower than before blood transfusion (*p *< 0.05), but the difference between groups 30 min after blood transfusion was not statistically significant (*p *> 0.05); at the 24- h postoperative period, no statistical difference was found during the comparison of HCT, Hb, PLT, and RBC in all three groups (*p *> 0.05). This shows that both autologous and allogeneic blood transfusion can effectively replenish blood volume and promote the body’s circulatory function to return to normal. The data also showed that SpO_2_ was essentially within the normal range before and after transfusion in all three groups of patients. This may be related to the fact that none of the patients included in this group had Hb below 80 g/L, despite the presence of hemorrhage, and, therefore, did not result in significant tissue hypoxia (21). Our data also showed that there was no statistical difference when Na^+^, K^+^, and Cl^−^ were compared among the three groups at the 24- h postoperative period (*p* > 0.05). This may be related to the fact that most of the blood transfusions in this group were component transfusions in smaller amounts, which did not cause electrolyte disturbances.

## Conclusion

In conclusion, the salvage autologous blood transfusion technique for treating ectopic pregnancy hemorrhage is a safe and feasible method to rescue patients with such condition. Although it cannot completely replace the allogeneic blood transfusion technique, it can rapidly replenish the patient’s blood volume and save blood resources without causing postoperative hemodynamic, routine blood, and electrolyte abnormalities.

## Data Availability

All data in the submitted article used or analyzed can be obtained from the respective authors.
